# ‘nparACT’ package for R: A free software tool for the non-parametric analysis of actigraphy data

**DOI:** 10.1016/j.mex.2016.05.006

**Published:** 2016-05-24

**Authors:** Christine Blume, Nayantara Santhi, Manuel Schabus

**Affiliations:** a Centre for Cognitive Neuroscience Salzburg (CCNS), University of Salzburg, Hellbrunner Strasse 34, 5020 Salzburg, Austria; b Laboratory for Sleep, Cognition and Consciousness Research, University of Salzburg, Hellbrunner Strasse 34, 5020 Salzburg, Austria; c Surrey Sleep Research Centre, Faculty of Health and Medical Sciences, University of Surrey, Egerton Road, Guildford GU2 7XP, United Kingdom

**Keywords:** Actigraphy, R, Sleep, Circadian rhythm, Zeitgeber, Internal clock, Amplitude

## Abstract

For many studies, participants’ sleep-wake patterns are monitored and recorded prior to, during and following an experimental or clinical intervention using actigraphy, i.e. the recording of data generated by movements. Often, these data are merely inspected visually without computation of descriptive parameters, in part due to the lack of user-friendly software. To address this deficit, we developed a package for R Core Team [6], that allows computing several non-parametric measures from actigraphy data. Specifically, it computes the interdaily stability (IS), intradaily variability (IV) and relative amplitude (RA) of activity and gives the start times and average activity values of M10 (i.e. the ten hours with maximal activity) and L5 (i.e. the five hours with least activity). Two functions compute these ‘classical’ parameters and handle either single or multiple files. Two other functions additionally allow computing an L-value (i.e. the least activity value) for a user-defined time span termed ‘Lflex’ value. A plotting option is included in all functions. The package can be downloaded from the Comprehensive R Archives Network (CRAN).

• The package ‘nparACT’ for R serves the non-parametric analysis of actigraphy data.
• Computed parameters include interdaily stability (IS), intradaily variability (IV) and relative amplitude (RA) as well as start times and average activity during the 10 h with maximal and the 5 h with minimal activity (i.e. M10 and L5).

## Method details

Actigraphy is a non-invasive method of monitoring participants’ rest-activity cycles, which is most commonly used in sleep and circadian rhythm research [2], [8]. In many studies, actigraphy recordings are used to ensure that participants adhere to a prescribed sleep-wake rhythm (cf. e.g. [5], [9]). Moreover, actigraphy data can inform about disturbances of the sleep-wake cycle such as circadian rhythm disorders and sleep disorders (for an overview see [1]). It is measured with a wrist-watch-like device, usually worn on the non-dominant hand, for several days before, during and after an experiment. In some cases ankle movements rather than wrist movements are expected to reflect the ‘true’ activity of the participant (e.g. when the upper limbs are spastic) and in these instances the device can be worn on the ankle. Movements the device undergoes are continuously recorded with a previously specified sampling rate (SR; e.g. 4/60 Hz).

In most studies, however, the analysis of actigraphy data is limited to a rather crude visual inspection of the general pattern of rest and activity or sleep and wakefulness, which is also reflected in the purposes other available R packages serve. The ‘PhysicalActivity’ package [3] for example only allows analysing device wear and non-wear time intervals and other tools such as the ‘accelerometry’ package [10] even focus solely on the analysis of periods of activity thus completely ignoring rhythmic changes between rest and activity. We think that sometimes, however, a more advanced analysis of rest-activity cycles could yield further information and certain parameters may even become variables of interest. While the ‘GGIR’ package computes the M5 and L5 descriptives (i.e. five hours with minimal and maximal activity) for data obtained with specific devices, it does not calculate other parameters that might be valuable for a comprehensive description of the rest-activity pattern. In particular, parameters quantifying how well the period length of a rhythm matches the earth’s 24 h light-dark cycle, how fragmented a rhythm is and what amplitude the rest-activity pattern has could be of special interest. We assume that the reluctance to further analyse actigraphy data regarding such parameters and thus the underestimation of their scientific value is, partly, due to the lack of adequate analysis tools. We thus developed the package ‘nparACT’ for R Core Team [6], that computes several non-parametric measures from actigraphy data that allow for a quantification of the parameters mentioned above (cf. [11], [12], [13]). The most recent version of the package can be downloaded from the Comprehensive R Archives Network (CRAN) and is, just as R itself, open source. As the package is updated from time to time, we deliberately do not provide a zip file of the package along with this publication.

## Method description

### Data requirements

Confounding factors such as the dependence of rest-activity patterns on the day of the week can impair the interpretability of the data. Actigraphy data should therefore be acquired over the course of a whole week or multiple weeks, unless, of course, workdays are of special interest. In repeated measures designs the same days of the week should be recorded on each occasion as otherwise the interpretability of the results may suffer. We moreover recommend recordings to cover at least five workdays or a whole week to obtain reliable estimates of the parameters (cf. also [12]). As participants do usually not wear the actigraph continuously during the recording period (e.g. they take it off for showering or exercising), recordings typically contain ‘invalid’ data. Although the ‘nparACT’ package does not offer an option to exclude these periods and impute the respective values, users are advised to consider this as an additional step during preparation of the data for further analysis using ‘nparACT’.

Following recording, data are usually transferred from the device to a computer using manufacturer-specific software. For devices that sample at a high frequency (e.g. 60 Hz), data can normally be downsampled within this software. The ‘nparACT’ package can handle any type of sampling rate and will downsample data to 1/60 Hz if necessary, but data files with lower input sampling rates will speed up the analyses. Besides this, most devices nowadays record movements in three dimensions and software provided by the manufacturer usually includes an option to integrate the three axes into one activity value. This must be done prior to processing data within the package. Finally, the input file must contain one column with date and/or time and one column with actigraphy values. The former must be of form “YYYY-MM-DD HH:MM:SS” or “YYYY/MM/DD HH:MM:SS” (i.e. an unambiguous date format). Date and/or time must be in the first, activity values in the second column. For the data structure please also see the ‘sleepstudy’ example data that comes with the package. If one of the functions for the analysis of multiple files is used, data must be text files or comma separated values (CSV) files. This is not necessary for analysing single actigraphy files as in this case the file has to be loaded into the R environment before functions can be applied in a separate step.

### Preprocessing options

Analyses should be based on data spanning full days only as interdaily stability (IS) and intradaily variability (IV) depend on the day/night ratio. Data are cut to full days if the *fullday* argument is set to TRUE, which is the default. Furthermore, in some cases it may be desirable to decrease the sensitivity of activity detection post hoc. While this requires caution as well as a careful consideration of pros and cons, we have included an option to define a cut-off value below which all activity values are set to zero. The default value of the *cutoff* is 1, in which case all values are taken into account as they are.

### Non-parametric actigraphy measures

The package computes the interdaily stability (IS), intradaily variability (IV) and relative amplitude (RA) of activity values. Moreover, it gives the start times and average activity of M10 (i.e. 10 h with maximal activity) and L5 (i.e. five hours with least activity).

a) Interdaily stability
  IS quantifies the stability of rest-activity rhythms or the invariability of the rhythm between different days. It, thus, describes the coupling of the rhythm to external zeitgebers, that is environmental cues such as light that entrain an organism’s internal biological clock to the earth’s 24 h cycle. IS varies between 0 (Gaussian noise) and 1 with values closer to 1 indicating stronger coupling to a zeitgeber with a period length of 24 h. Among various zeitgebers, sunlight is thought to be the strongest one, but also social factors such as worktimes, nutrition and maybe even sleep itself have been suggested to influence the rhythm [4], [7]. It is computed according to the following formula.
  (1) $$IS=\frac{n\sum_{h=1}^{p}{\left({\bar{X}}_{h}-\bar{X}\right)}^{2}}{p\sum_{i=1}^{n}{\left(X_{i}-\bar{X}\right)}^{2}}$$
  Formula (1): Interdaily stability (IS). *n* is the total number of sampling points and *p* is the number of sampling points per day while ${\bar{x}}_{h}$ are the hourly means, $\bar{x}$ is the grand average of all data and $x_{i}$ denotes the activity value from each sampling point. Please note that *x_i_* are hourly values in ‘nparACT’.
b) Intradaily variability
  In contrast to IS, IV quantifies the fragmentation of a rest-activity pattern. IV converges to zero for a perfect sine wave and approaches two for Gaussian noise. It may even be higher than two if a definite ultradian component with a period length of two hours is present in the rest-activity cycle. IV is computed according to the following formula.
  (2) $$IV=\frac{n\sum_{i=2}^{n}{\left(X_{i}-X_{i-1}\right)}^{2}}{\left(n-1\right)\sum_{i=1}^{n}{\left(X_{i}-\bar{X}\right)}^{2}}$$
  Formula (2): Intradaily variability (IV). *n* is the total number of sampling points and *p* is the number of sampling points per day $\bar{x}$ is the grand average of all data and $x_{i}$ denotes the activity value from each sampling point. Please note that *x_i_* are hourly values in nparACT.
c) Relative amplitude
  RA is a non-parametric parameter, which can be calculated from the M10 and L5 values, that is the ten hours with maximal (M10) and the five hours with minimal (L5) activity. Usually, M10 covers 10 h during the day and may be influenced by e.g. daytime napping. L5, on the other hand, should reflect movements during the night as well as arousals and awakenings. The formula for the computation of RA is:
  (3) $$RA=\frac{\left(M10-L5\right)}{\left(M10+L5\right)}$$
  Formula (3): Relative amplitude (RA). *M10* is the averaged activity of the ten consecutive hours with maximal activity, *L5* is the averaged activity of the five consecutive hours with minimal activity
  To find M10 and L5 averages for ten and five hours are calculated on a minute-wise level across days. More precisely, minute-wise averages are first calculated across days. Subsequently, averages for five and 10 h are computed (e.g. for L5 from 6:00 to 11:00, from 6:01 to 11:01 and so on) across 24 h yielding a total of 1440 values. From these, the maximal value from the 10 h averages and the minimal value from the five hour averages are picked as well as the times of the day when M10 and L5 start.
d) Lflex

In the package, we additionally included the possibility to find the lowest activity during a user-defined time window, termed the *Lflex* value. This way one could, for instance, look for the 7.5 h with least activity in a group of insomniacs and see whether their average activity levels during this time or whether the start time deviates from a healthy control group.

### Functions

The package comprises two function families besides several auxiliary functions, which may be used to customise the package by experienced R users. The ‘base’ family, (i.e. ‘nparACT_base’ and the ‘nparACT_base_loop’) computes the classic non-parametric actigraphy measures IS, IV, RA and the M10 and L5 values and their start times. The ‘flex’ family (i.e. ‘nparACT_flex’ and ‘nparACT_flex_loop’) additionally computes the *Lflex* value for a user-defined time window. Both families each comprise one function that is suitable for the analysis of a single actigraphy file and one function that loops though multiple files. All functions give a result matrix containing the parameters computed.

### Plots

Users can choose whether they would like to receive plots by setting the plotting parameter of the function to *plot* *=* *TRUE* (default) or *plot* *=* *FALSE*. For single files, (a) a classic dual-day display plot, (b) a plot of minute-wise averages of activity across 24 h as well as a (c) plot for hourly averages across 24 h is produced.

**Fig. 1:**
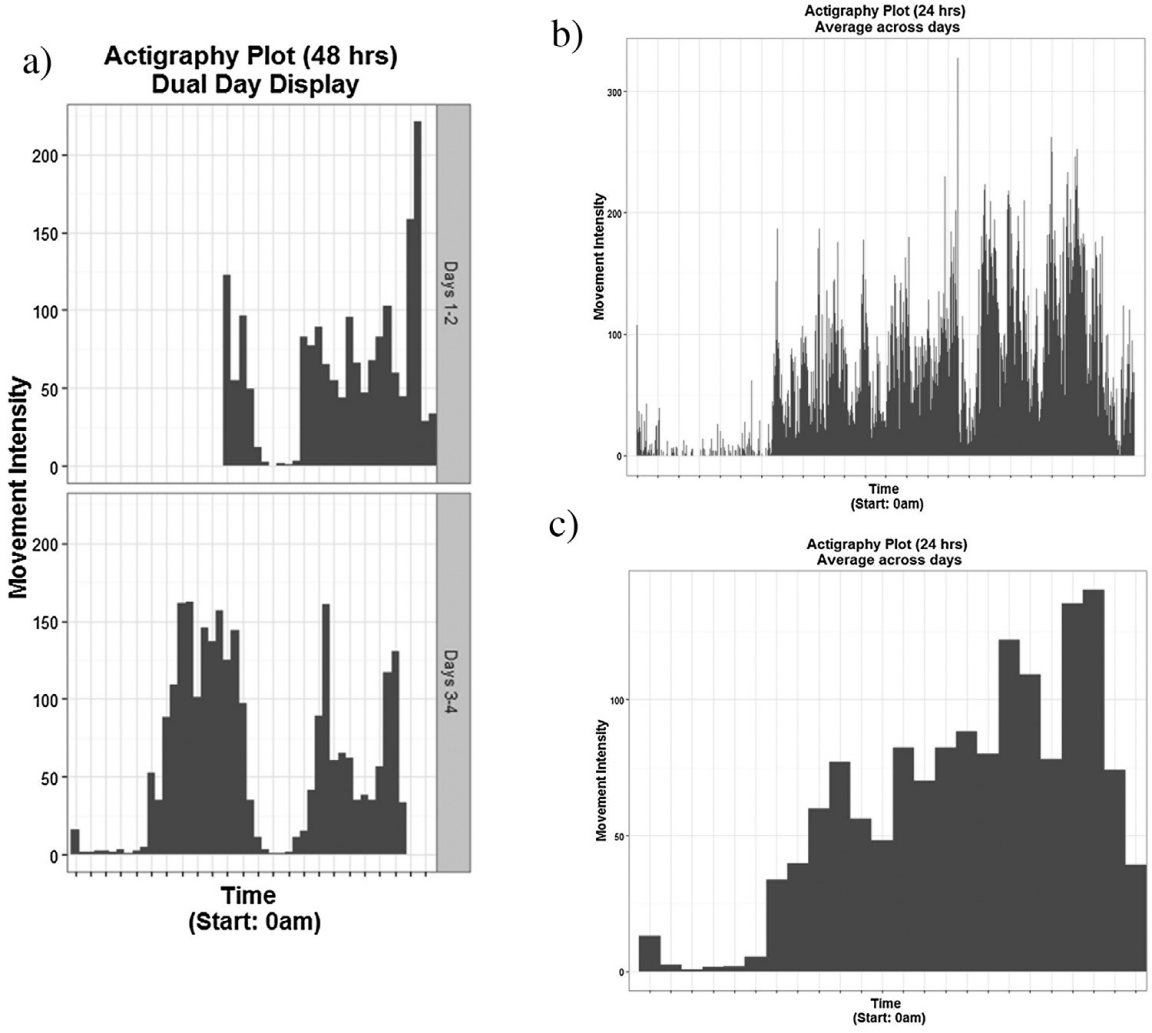
Plots for single actigraphy files. (a) Classic dual-day display plot, (b) plot of minute-wise averages of activity across 24 h, (c) plot for hourly averages across 24 h.

For multiple files only the grand average of hourly activity values across 24 h are plotted similarly to Fig. 1c.

## Conflict of interest

The authors declare no conflict of interest.
